# Thalamomesencephalic cavernoma: anterior transcallosal transchoroidal approach

**DOI:** 10.3171/2019.7.FocusVid.191213

**Published:** 2019-07-01

**Authors:** Francesco Certo, Giada Toccaceli, Roberto Altieri, Giuseppe M. V. Barbagallo

**Affiliations:** Department of Neurological Surgery, Policlinico “G. Rodolico” University Hospital, Catania, Italy

**Keywords:** cavernoma, third ventricle, transcallosal, approach, video

## Abstract

We present a case of a 62-year-old man with acute onset of diplopia, headache, and vomiting for a bleeding thalamomesencephalic cavernoma. The lesion was removed via the anterior transcallosal transchoroidal approach. His head was slightly flexed and a right paramedian craniotomy for an interhemispheric approach was performed. The interhemispheric fissure was split and, after callosotomy, the choroidal fissure was opened along the tenia fornicis to enter the velum interpositum and enlarge the foramen of Monro. The cavernoma was then identified and resected. There were no long-term postoperative neurological deficits. This approach is a valid alternative for thalamomesencephalic lesions.

The video can be found here: https://youtu.be/DJdorbzDnH0.

## Transcript

We present a case of thalamomesencephalic cavernoma removed by anterior transcallosal transchoroidal approach.

The patient is a 62-year-old man who presented, after mild brain injury, acute onset of diplopia, headache, and vomiting. Because of persistence of symptoms, he performed a brain CT that showed a right thalamomesencephalic bleeding, with a maximum diameter of 12 mm.

For these reasons, patient was admitted in Neurosurgery Department and performed an MRI scan that showed a right periaqueductal gray mesencephalic and thalamic cavernoma and the presence of a minimal intraventricular bleeding.

1:29 Mesencephalic cavernous malformations (Lee and Lui, 1996; Garrett and Spetzler, 2009; Beechar et al., 2017; Ren et al., 2017) are usually approached via:

### Anterior approaches (ventral cavernomas)

• Orbitozygomatic or frontotemporal-orbitozygomatic approaches
• Subtemporal approach
• Pterional transsylvian approach

### Posterior approaches (dorsal cavernomas)

• Supracerebellar infratentorial approach
• Occipital transtentorial/interhemispheric

### Superior approaches (ventral cavernomas surfacing the floor of third ventricle)

• Transcallosal transchoroidal
• Transcallosal transforaminal

For the reported case, an anterior route should be advised (OZ, FTOZ, pterional), but these approaches are associated with increased risks of:

• Pyramidal tract injury
• Mechanical or vascular injuries

Superior surface of cavernoma was directly visible in the context of the floor of third ventricle.

Due to the symptomatic onset with bleeding, we decide to operate the patient and we choose the anterior transcallosal transchoroidal approach.

The patient was in anti-Trendelenburg supine position with the head slightly flexed. We performed a C-shaped skin incision and the craniotomy is tailored with respect to the angle of approach.

We used, as surgical tools, intraoperative CT, neuronavigation, and neurophysiological monitoring.

In this image we can see both the AxiEM neuronavigation and the radiolucent Mayfield head holder, useful to perform the intraoperative CT scan.

After coagulating and dissecting some bridging veins we proceeded to open the interhemispheric fissure, detect and separate pericallosal arteries, and find the corpum callosum.

3:18 In this phase, neuronavigation is useful to be guided in the surgical trajectory.

3:21 After callosotomy we could enter in the right lateral ventricle, opening a wide fissure.

3:38 The dissection of the choroidal fissure to release the choroid plexus from the fornix must be performed extremely carefully.

So, having identified the choroid plexus, the thalamostriate veins, and, anteriorly, the Monro foramen, we proceeded to a careful dissection of choroidal fissure, to release the choroid plexus from the fornix, avoiding any kind of injury.

4:03 Identification of thalamostriate veins and foramen of Monro

The choroidal fissure was opened along the tenia fornicea to enter the velum interpositum and enlarge the foramen of Monro

We entered in the third ventricle and we detected the cavernoma. We checked the position with the neuronavigation and then we proceded to remove it.

4:37 Excision of cavernoma

In this phase neuromonitoring is useful to avoid neurological damages.

5:35 Hemostasis

6:16 Watertight dural closure

Postoperative neuroimaging showed the complete resection of cavernoma with minimal cerebral manipulation.

In conclusion, thalamomesencephalic cavernomas continue to represent a considerable microsurgical challenge. Even if most ventral thalamomesencephalic cavernomas can be removed via transylvian, subtemporal, or frontotemporal-zygomatic approach, the anterior transcallosal transchoroidal approach can serve as valuable alternative surgical corridor.
